# Effect of marital status on the survival of patients with hepatocellular carcinoma treated with surgical resection: an analysis of 13,408 patients in the surveillance, epidemiology, and end results (SEER) database

**DOI:** 10.18632/oncotarget.12722

**Published:** 2016-10-18

**Authors:** Chao Wu, Ping Chen, Jian-Jun Qian, Sheng-Jie Jin, Jie Yao, Xiao-Dong Wang, Dou-Sheng Bai, Guo-Qing Jiang

**Affiliations:** ^1^ Department of General Surgery, Wuxi 9th People's Hospital, Wuxi, China; ^2^ Department of Hepatobiliary and Pancreatic Surgery, Clinical Medical College of Yangzhou University, Yangzhou, China

**Keywords:** hepatocellular carcinoma, marital status, SEER, survival analysis, surgery

## Abstract

Marital status has been reported as an independent prognostic factor for survival in various cancers, but it has been rarely studied in hepatocellular carcinoma (HCC) treated by surgical resection. We retrospectively investigated Surveillance, Epidemiology, and End Results (SEER) population-based data and identified 13,408 cases of HCC with surgical treatment between 1998 and 2013. The patients were categorized according to marital status, as “married,” “never married,” “widowed,” or “divorced/separated.” The 5-year HCC cause-specific survival (HCSS) data were obtained, and Kaplan–Meier methods and multivariate Cox regression models were used to ascertain whether marital status is also an independent prognostic factor for survival in HCC. Patients in the widowed group had the higher proportion of women, a greater proportion of older (>60 years) patients, more frequency in latest year of diagnosis (2008-2013), a greater number of tumors at TNM stage I/II, and more prevalence at localized SEER Stage, all of which were statistically significant within-group comparisons (*P* < 0.001). Marital status was demonstrated to be an independent prognostic factor by multivariate survival analysis (*P* < 0.001). Married patients had better 5-year HCSS than did unmarried patients (46.7% vs 37.8%) (*P* < 0.001); conversely, widowed patients had lowest HCSS compared with all other patients, overall, at each SEER stage, and for different tumor sizes. Marital status is an important prognostic factor for survival in patients with HCC treated with surgical resection. Widowed patients have the highest risk of death compared with other groups.

## INTRODUCTION

Worldwide, primary liver cancer (LC), which consists of primary hepatocellular carcinoma (HCC), intrahepatic cholangiocarcinoma (ICC), and combined HCC and ICC, is the fifth most common cancer and the third leading cause of cancer-associated mortality [1]. In Western countries, the rise of HCC has been linked with increasing hepatitis C infection and alcohol consumption [2]; however, the incidence of LC has also been rapidly increasing in Asian countries, such that the incidence among Asians is now twice that among Africans [3].

Extensive research has demonstrated that marital status is an independent prognostic factor of survival in several cancers [4–7]. Li et al reported that unmarried patients with colorectal cancer were at greater risk for cancer-specific mortality and that widowed patients were at highest risk for death compared with other groups [6]. Wang et al showed that marital status was an important prognostic factor in pancreatic cancer and that widowed patients were at the greatest risk for death [7]. A study reported, in primary liver cancer patients, married patients enjoyed survival benefits while widowed persons suffered survival disadvantages in both overall survival and cancer-specific survival [8]. LC is a heterogenous cancer. As we know, HCC and ICC may have different pathogenesis and different biological behavior, or even different long-term survivals. So analysis on survivals of HCC and ICC separately might be more meaningful and reasonable. Yet, the effect of marital status on HCC survival with surgical resection has not been rigorously studied. Therefore, the aim of this study was to explore the relationship between marital status and HCC outcomes, as well as the potential underlying mechanisms. We extracted data from the Surveillance, Epidemiology, and End Results (SEER) cancer registry to investigate the effect of marital status on HCC cause-specific survival (HCSS) in patients with HCC treated by surgical resection.

## RESULTS

### Baseline patient characteristics

A total of 13,408 eligible patients were identified during the 15-year study period (between 1998 and 2013), including 10,071 male and 3,337 female patients. Of these, 966 (7.2%) were widowed, 8494 (63.4%) were married, 2265 (16.9%) had never married. The 200 (1.5%) individuals who were separated and 1,483 (11.1%) who were divorced were grouped together in the divorced/separated group in our study. Patients in the widowed group had the higher proportion (62.4%) of women within-group comparisons, a greater proportion (84.6%) of older (>60 years) patients, more frequency (49.1%) in latest years of diagnosis (2008-2013), a greater number (29.8%) of TNM stage I/II tumors, and more prevalence (78.1%) at localized SEER Stage, all of which were statistically significant (*P* < 0.001). The baseline patient demographics and tumor characteristics are described in Table 1.

**Table 1:** Baseline demographic and tumor characteristics of patients in SEER database

Characteristic	Total	Widowed	Married	Never married	Divorced/Separated	*P*
	(n = 13408) *N* (%)	(n = 966) *N* (%)	(n = 8494) *N* (%)	(n = 2265) *N* (%)	(n = 1683) *N* (%)	
Sex						< 0.001
Male	10071 (75.1)	363(37.6)	6743 (79.4)	1749 (77.2)	1216 (72.3)	
Female	3337 (24.9)	603 (62.4)	1751 (20.6)	516 (22.8)	467 (27.7)	
Age						< 0.001
≤60	7043 (52.5)	149 (15.4)	4307 (50.7)	1556 (68.7)	1031 (61.3)	
>60	6365 (47.5)	817 (84.6)	4187 (49.3)	709 (31.3)	652 (38.7)	
Race						< 0.001
White	8750 (65.3)	628 (65.0)	5387 (63.4)	1494 (66.0)	1241 (73.7)	
Black	1412 (10.5)	95 (9.8)	647 (7.6)	451 (19.9)	219 (13.0)	
Other*	3246 (24.2)	243 (25.2)	2460 (29.0)	320 (14.1)	223 (13.3)	
Year of diagnosis						< 0.001
1998-2002	2029 (15.1)	177 (18.3)	1380 (16.2)	262 (11.6)	210 (12.5)	
2003-2007	4806 (35.8)	315 (32.6)	3117 (36.7)	768 (33.9)	606 (36.0)	
2008-2013	6573 (49.0)	474 (49.1)	3997 (47.1)	1235 (54.5)	867 (51.5)	
Pathological grading						< 0.001
Well/Moderate	6708 (50.0)	478 (49.5)	4376 (51.5)	1076 (47.5)	778 (46.2)	
Poor/Anaplastic	1601 (11.9)	115 (11.9)	1054 (12.4)	247 (10.9)	185 (11.0)	
Unknown	5099 (38.0)	373 (38.6)	3064 (36.1)	942 (41.6)	720 (42.8)	
TNM Stage						< 0.001
I/II	3708 (27.7)	288 (29.8)	2230 (26.3)	704 (31.1)	486 (28.9)	
III/IV	593 (4.4)	42 (4.3)	365 (4.3)	121 (5.3)	65 (3.9)	
Unknown	9107 (67.9)	636 (65.8)	5899 (69.4)	1440 (63.6)	1132 (67.3)	
Tumor Size						
<3 cm	4033 (30.1)	254 (26.3)	2444 (28.8)	752 (33.2)	583 (34.6)	< 0.001
3–5 cm	3289 (24.5)	218 (22.6)	2056 (24.2)	555 (24.5)	460 (27.3)	
>5 cm	2715 (20.2)	206 (21.3)	1775 (20.9)	465 (20.5)	269 (16.0)	
Not stated	3371 (25.1)	288 (29.8)	2219 (26.1)	493 (21.8)	371 (22.0)	
SEER Stage						0.268
Localized	9941 (74.1)	754 (78.1)	6280 (73.9)	1666 (73.6)	1241 (73.7)	
Regional	2617 (19.5)	153 (15.8)	1678 (19.8)	452 (20.0)	334 (19.8)	
Distant	514 (3.8)	32 (3.3)	327(3.8)	92 (4.1)	63 (3.7)	
Unstaged	336 (2.5)	27 (2.8)	209 (2.5)	55 (2.4)	45 (2.7)	

Abbreviations: SEER, Surveillance, Epidemiology, and End Results.

* Other includes American Indian/Alaska native, Asian/Pacific Islander, and unknown.

### Effect of marital status on HCSS

**Figure 1:**
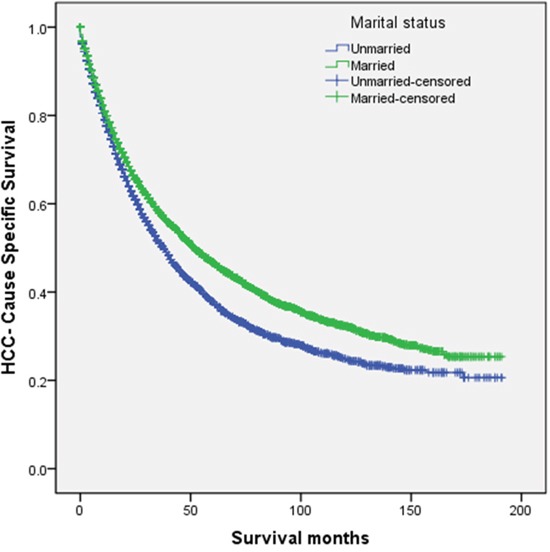
Survival curves in hepatocellular carcinoma patients treated with surgical resection between the unmarried patients and the married patients χ2 = 68.610, P < 0.001.

**Figure 2:**
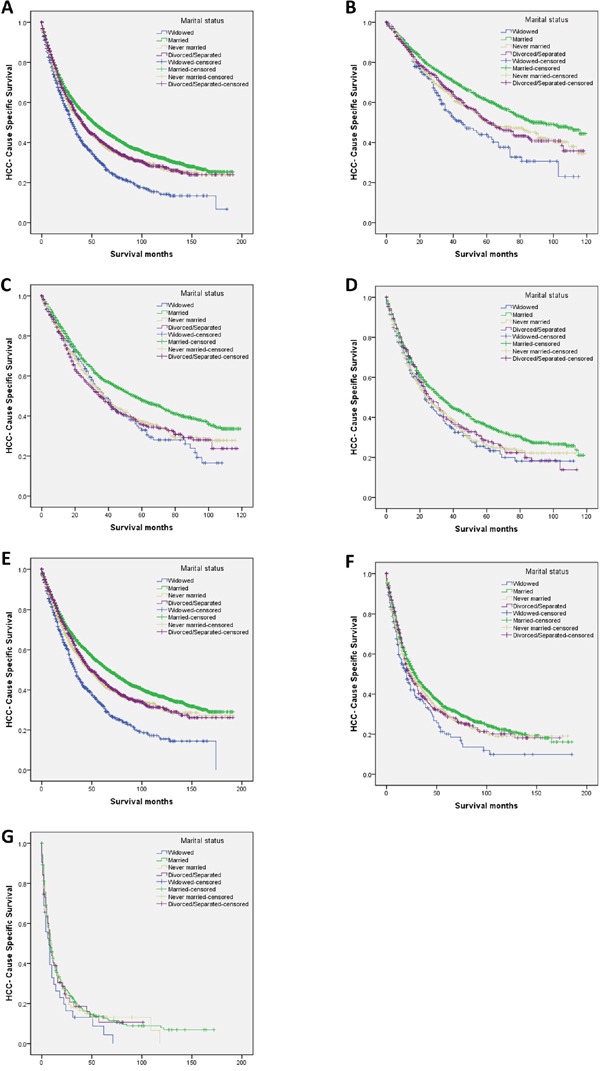
Survival curves in hepatocellular carcinoma patients according to marital status **A.** Overall: χ^2^ = 109.915 (*P* < 0.001); **B.** tumor diameter <3 cm: χ^2^ = 35.696 (*P* < 0.001); **C.** tumor size 3–5 cm: χ^2^ = 31.884 (*P* < 0.001); **D.** tumor diameter >5 cm: χ^2^ = 16.972 (*P* = 0.004); **E.** SEER localized stage: χ^2^ = 119.747 (*P* < 0.001); **F.** SEER regional stage: χ^2^ = 11.960 (*P* = 0.008); and **G.** SEER distant stage: χ^2^ = 2.743 (*P* = 0.433). SEER: Surveillance, Epidemiology, and End Results.

Married patients had better 5-year HCSS than did unmarried patients (46.7% vs 37.8%) (*P* < 0.001) (Figure 1). The 5-year HCSS was 29.4% in the widowed group, 46.7% in the married group, 39.4% in the never married group, and 40.4% in the divorced/separated group, all significantly different according to the univariate log rank test (*P* < 0.001) (Figure 2A). Older age (*P* < 0.001), black race (*P* < 0.001), poor or undifferentiated pathology grade (*P* < 0.001), the latest year of diagnosis (*P* < 0.001), TNM stage III/IV disease (*P* < 0.001), tumor size >5 cm (*P* < 0.001), and SEER distant stage (*P* < 0.001) were regarded as significant risk factors for poor survival on univariate analysis (Table 2). When multivariate analysis with Cox regression was performed, all the aforementioned variables were validated as independent prognostic factors for poor survival (Table 2), as follows: age (>60 years, hazard ratio [HR] 1.399, 95% confidence interval [CI] 1.332–1.469), race (black, HR 1.257, 95% CI 1.167–1.353; other races, HR 0.818, 95% CI 0.773–0.867), year of diagnosis (2003-2007, HR 0.997, 95% CI 0.915–1.086; 2008-2013, HR 0.827, 95% CI 0.748–0.916), pathological grading (poor or undifferentiated tumor, HR 1.458, 95% CI 1.356–1.568, unknown pathology grade, HR 1.386, 95% CI 1.317–1.459), TNM stage (stage III/IV, HR 1.327, 95% CI 1.210–1.455; unknown stage, HR 1.129, 95% CI 0.997–1.278), tumor size (3–5 cm tumor, HR 1.411, 95% CI 1.312–1.516; >5 cm tumor, HR 1.632, 95% CI 1.504–1.771; unstated tumor size, HR 1.456, 95% CI 1.281–1.655), SEER Stage (regional stage, HR 1.427, 95% CI 1.340–1.518; distant stage, HR 2.448, 95% CI 2.188–2.739; unstaged, HR 1.052, 95% CI 0.911–1.216), marital status (married, HR 0.745, 95% CI 0.683–0.812; never married, HR 0.893, 95% CI 0.807–0.988; divorced/separated, HR 0.862, 95% CI 0.776–0.957).

**Table 2:** Univariate and multivariate survival analysis for evaluating the influence of marital status on HCC cause-specific survival in SEER database

Variable	Total	5-year CCS	Univariate analysis		Multivariate analysis	
	13408		Log rank χ2 test	*P*	HR (95%CI)	*P*
Sex			0.010	0.921		NI
Male	10071	43.5%				
Female	3337	43.5%				
Age			204.008	< 0.001		< 0.001
≤60	7043	49.3%			Reference	
>60	6365	36.5%			1.399 (1.332–1.469)	
Race			83.046	< 0.001		< 0.001
White	8750	43.8%			Reference	
Black	1412	33.0%			1.257 (1.167–1.353)	< 0.001
Other*	3246	47.1%			0.818 (0.773–0.867)	< 0.001
Year of diagnosis			125.996	< 0.001		< 0.001
1998-2002	2029	37.6%			Reference	
2003-2007	4806	41.9%			0.997 (0.915–1.086)	0.937
2008-2013	6573	46.4%			0.827 (0.748–0.916)	< 0.001
Pathological grading			266.354	< 0.001		< 0.001
Well/Moderate	6708	50.4%			Reference	
Poor/Anaplastic	1601	33.9%			1.458 (1.356–1.568)	< 0.001
Unknown	5099	37.3%			1.386 (1.317–1.459)	< 0.001
TNM Stage			262.301	< 0.001		< 0.001
I/II	3708	64.8%^#^			Reference	
III/IV	593	37.2%^#^			1.327 (1.210–1.455)	< 0.001
Unknown	9107	53.3%^#^			1.129 (0.997–1.278)	0.056
Tumor Size			495.989	< 0.001		< 0.001
<3 cm	4033	56.7%			Reference	
3–5 cm	3289	43.4%			1.411 (1.312–1.516)	< 0.001
>5 cm	2715	32.6%			1.632 (1.504–1.771)	< 0.001
Not stated	3371	37.7%			1.456 (1.281–1.655)	< 0.001
SEER Stage			919.645	< 0.001		< 0.001
Localized	9941	48.7%			Reference	
Regional	2617	31.3%			1.427 (1.340–1.518)	< 0.001
Distant	514	12.9%			2.448 (2.188–2.739)	< 0.001
Unstaged	336	36.1%			1.052 (0.911–1.216)	0.489
Marital Status			109.915	< 0.001		< 0.001
Widowed	966	29.4%			Reference	
Married	8494	46.7%			0.745 (0.683–0.812)	< 0.001
Never married	2265	39.4%			0.893 (0.807–0.988)	0.028
Divorced/Separated	1683	40.4%			0.862 (0.776–0.957)	0.005

Abbreviations: HCC, hepatocellular carcinoma; SEER, Surveillance, Epidemiology, and End Results; CCS, cause-specific survival.

* Other includes American Indian/Alaska native, Asian/Pacific Islander, and unknown.

# 3-year CCS. Because TNM stage record according to the AJCC Cancer Staging Manual (7th edition) in the SEER database began from 2009, and ended at 2013, its 5-year CCS did not exist.

NI: not included in the multivariate survival analysis.

### Subgroup analysis of the effect of marital status, according to tumor size

We further analyzed the effects of marital status on survival among tumors of different sizes, and we observed three interesting findings: First, tumor size was an independent factor for poor survival, both in univariate and multivariate analysis (*P* < 0.001). Second, widowed patients had the lowest survival rate in comparisons at all tumor sizes: Widowed patients had a 17.1% reduction in 5-year LCSS compared with married patients for tumors with diameter <3 cm (44.0% *vs* 61.1%) (*P* < 0.001), a 14.9% reduction for tumors with diameter 3–5 cm (32.9% *vs* 47.8%) (*P* < 0.001), and a 11.2% reduction for tumors with diameter >5 cm (24.5% *vs* 35.7%) (*P* < 0.001). Third, there were only very small difference in survival between the never married and divorced/separated patients for any tumor size: At tumor size <3 cm and >5 cm respectively, patients in the never married group had a 0.6% and 2.4% decrease in 5-year HCSS compared with patients in the divorced/separated group; at tumor size 3–5 cm, the never married group showed a 1.4% increase in 5-year HCSS (Table 3, Figure 2B–2D).

**Table 3:** Univariate and multivariate analysis of marital status on HCC cause-specific survival based on different tumor size

Variable	Total	5-year CCS	Univariate analysis		Multivariate analysis	
			Log rank χ2 test	*P*	HR(95% CI)	*P*
**Tumor Size**						
**<3 cm**	4033					
**Marital status**			35.696	< 0.001		< 0.001
Widowed	254	44.0%	Reference		Reference	
Married	2444	61.1%	22.809	< 0.001	0.612 (0.499–0.750)	< 0.001
Never married	752	50.4%	4.322	0.038	0.792 (0.633–0.991)	0.041
Divorced/Separated	583	51.0%	4.680	0.031	0.786 (0.624–0.991)	0.041
**3–5 cm**	3289					
**Marital status**			31.884	< 0.001		< 0.001
Widowed	218	32.9%	Reference		Reference	
Married	2056	47.8%	11.353	< 0.001	0.725 (0.600–0.877)	< 0.001
Never married	555	37.3%	0.425	0.514	0.933 (0.754–1.155)	0.527
Divorced/Separated	460	35.9%	0.026	0.871	0.981 (0.790–1.219)	0.863
**>5 cm**	2715					
**Marital status**			16.972	< 0.001		< 0.001
Widowed	206	24.5%			Reference	
Married	1775	35.7%	8.015	0.005	0.770 (0.642–0.924)	0.005
Never married	465	26.2%	0.259	0.611	0.947 (0.770–1.164)	0.603
Divorced/Separated	269	28.6%	0.709	0.400	0.913 (0.727–1.146)	0.432

Abbreviations: HCC, hepatocellular carcinoma; CCS, cause-specific survival.

### Subgroup analysis of the effect of marital status, according to SEER stage

We also analyzed the effects of marital status on survival at each SEER stage. There were no significant differences in the distribution of SEER stages among the different marital status groups (Table 1). Again, we had three interesting findings: First, marital status was an independent risk factor for poor survival in patients with SEER localized and regional stage disease, both in univariate and multivariate analysis (*P* < 0.01); marital status was not significant at distant stage disease, in either univariate or multivariate analysis, possibly because of the smaller sample size (n=514)—in particular, there were only 32 widowed patients and 63 divorced/separated patients with distant stage disease. Second, widowed patients again had the lowest survival rate in comparisons at all tumor stages: Widowed patients had a 10.0% reduction in 2-year HCSS compared with married patients for localized stage tumors (62.0% *vs* 72.0%) (*P* < 0.001), a 12.0% reduction for regional stage tumors (42.1% *vs* 54.1%) (*P* = 0.001), and an 10.5% reduction for distant stage tumors (16.4% *vs* 26.9%) (*P* = 0.095). Third, there were almost no differences between the never married and divorced/separated patients for any SEER stage: For localized stage tumors, never married patients had a 3.0% decrease in 2-year HCSS compared with divorced/separated patients; for regional and distant stage tumors, respectively, never married patients had a 1.1% and 1.9% increase in survival compared with divorced/separated patients (Table 4, Figure 2E–2G).

**Table 4:** Univariate and multivariate analysis of marital status on HCC cause-specific survival based on different SEER Stage

Variable	Total	2-year CCS	Univariate analysis		Multivariate analysis	
			Log rank χ2 test	*P*	HR (95% CI)	*P*
**SEER Stage**						
**Localized**	9941					
**Marital status**			119.747	< 0.001		< 0.001
Widowed	754	62.0%	Reference		Reference	
Married	6280	72.0%	107.518	< 0.001	0.597 (0.541–0.659)	< 0.001
Never married	1666	67.6%	29.617	< 0.001	0.727 (0.648–0.816)	< 0.001
Divorced/Separated	1241	70.6%	36.034	< 0.001	0.700 (0.620–0.790)	< 0.001
**Regional**	2617					
**Marital status**			11.960	0.008		0.009
Widowed	153	42.1%	Reference		Reference	
Married	1678	54.1%	10.627	0.001	0.723 (0.593–0.881)	0.001
Never married	452	50.0%	4.197	0.040	0.795 (0.637–0.992)	0.042
Divorced/Separated	334	48.9%	4.166	0.041	0.789 (0.626–0.995)	0.045
**Distant**	514					
**Marital status**			2.743	0.433		0.464
Widowed	32	16.4%	Reference		Reference	
Married	327	26.9%	2.783	0.095	0.736 (0.505– 1.073)	0.111
Never married	92	24.7%	1.560	0.212	0.774 (0.508– 1.181)	0.235
Divorced/Separated	63	22.8%	1.230	0.267	0.768 (0.490– 1.204)	0.250

Abbreviations: HCC, hepatocellular carcinoma; SEER, Surveillance, Epidemiology, and End Results; CCS, cause-specific survival.

## DISCUSSION

Compared with those who have never married, separated, widowed, or divorced, married patients have longer overall survival and lower mortality for many major causes of death [9–11]. Using the SEER database to investigate the relationship between marital status and survival, the present study showed that in the context of HCC, widowed patients had significantly poorer HCSS than did their married counterparts. Furthermore, in multivariable analyses, the risk for widowed patients persisted even after adjusting for age, race, year of diagnosis, pathology grade, TNM stage, tumor size, and SEER Stage.

One hypothesis for the poor prognosis in unmarried individuals is delayed diagnosis with advanced tumor stage; however, as seen in Table 1, in our study group, however, the percentages of patients with localized and regional tumors or distant metastasis were comparable among the four subgroups. Moreover, widowed patients had the highest percentage of SEER localized stage disease. Widowed patients had worse 5-year HCSS compared with all other groups (all *P* < 0.001). Furthermore, among the patients at each SEER stage, the widowed group had worse 2-year HCSS compared with all other groups. Notably, at SEER distant stage, there was no significant difference in HCSS between the groups—this may have been the result of the smaller sample size, for which effects are more difficult to detect and/or to quantify (Table 4).

Our data also revealed that unmarried patients had a survival disadvantage that persisted for each different tumor size. In particular, widowed patients suffered from the poorest 5-year HCSS.

In attempting to explain the relationship between marital status and survival, psychosocial factors may provide a reasonable answer. Unmarried and especially, widowed patients may experience a lack of emotional and social support (otherwise provided by a spouse), and may display more distress, depression, and anxiety than do their married counterparts [12]. Additionally, marital status may affect the level of adherence to the treatment plan. Compared with their unmarried counterparts, married patients were shown to be more likely to comply with treatment, to seek treatment at more highly recognized centers, and to accept more aggressive treatment, all of which may make for better cancer control [13].

There is evidence that physiological changes accompanying stress and depression may worsen cancer outcome via different mechanisms. Decreased psychosocial support and increased psychological stress have been shown weaken immune function and in this way, may contribute to tumor progression and mortality [14–16]. Further, perceived lack of social support has been shown to decrease the activity of natural killer cells [17]. As well, chronic stress may cause a prolonged secretion of cortisol [18], which triggers a counterregulatory response in white blood cells, by downregulating their cortisol receptors. This downregulation also degrades the cellular response to anti-inflammatory signals and stimulates an increase in cytokine-mediated inflammatory processes [19], which, in colorectal cancer, has been confirmed to be a poor prognostic factor [20, 21]. Additionally, several other neuroendocrine mediators and cytokines present in depression and stress have been linked with an increased cancer metastasis [16]. Finally, depression and quality of life have been associated with an increased production of vascular endothelial growth factor, which may contribute to endothelial cell migration, proliferation, and proteolytic activity [22]. Accordingly, two meta-analyses confirmed that depression increased cancer mortality by 19% and 39%, respectively [23, 24].

This study analyzed data from the SEER database to add to current knowledge about the relationship between marital status and the postoperative prognosis of HCC; however, the study had several potential limitations. First, the SEER database only provided data on marital status at diagnosis. Whether the marital status varied during therapeutic process is unknown, but any variation would have affected the results. Second, some marital status data may have been inaccurate, for example, some patients classified as married may have actually separated, while other patients classified as never married may have been cohabitating. Third, the quality of the marriage including the “inner correlations” of marital status can also influence the survival of HCC patients—marital distress has also been linked with long-term immune consequences and has been associated with an increased risk of a variety of health problems [25]. Fourth, the SEER HCC database lacks quality data on adjuvant therapy, comorbidities, and recurrence. Finally, we hypothesized that psychosocial factors may have been important factors in the poor survival of unmarried patients, but we could not perform psychological tests to confirm our hypothesis.

Despite these potential limitations, our study results reconfirmed that unmarried patients are at greater risk for cancer-specific mortality. Furthermore, we showed that the unmarried patient groups were heterogeneous and that widowed patients were always at highest risk for death of cancer. Psychosocial factors may be the primary reasons for poor survival in unmarried patients. Therefore, physicians should include consideration of social supports during their care of unmarried patients with HCC and especially, of widowed patients, to help improve postoperative survival.

## MATERIALS AND METHODS

### Patient selection in the SEER database

The SEER program of the National Cancer Institute is an authoritative source of information on cancer incidence and survival in the United States. The SEER program registries routinely collect data on patient demographics, primary tumor site, the tumor morphology and stage at diagnosis, first course of treatment, and the follow up for survival—the SEER program is the only source of population-based information in the United States that includes the stage of cancer at the time of diagnosis as well as patient survival data.

Seer data contain no identifiers and have been widely used for studies of the relationship between marital status and survival outcome in patients with cancer [4, 5, 26–29]. We used SEER*Stat 8.1.5 software to identify patients with a histopathologic diagnosis of LC between 1998 and 2013. The morphology codes were limited to HCC (8170, 8171, 8172, 8173, 8174, 8175).

We excluded patients who were less than 18 years at diagnosis; did not undergo surgical resection for LC; had multiple primary cancers, of which the LC was not the first; and who had an unknown cause of death or unknown survival length.

According to the SEER staging system, tumors that remained in situ or confined to the organ of origin were considered to be localized; tumors that invaded locally or metastasized to regional lymph nodes were regarded as regional, while those that traveled to distant organs were considered to be distant.

### Statistical analysis

We analyzed sex, age, race, primary tumor site, pathology grade, histologic type, TNM stage, tumor size, SEER stage, and marital status at the time of diagnosis. The TNM stage was established according to the criteria described in the American Joint Committee on Cancer (AJCC) Cancer Staging Manual (7th edition). We classified patients as “married,” “never married,” “widowed,” or “separated/divorced.”

The primary endpoint of this study was HCSS, which was derived from the date of diagnosis and the date of cancer-specific death. Deaths attributed to HCC were treated as events, and deaths from other causes were treated as censored observations.

The baseline patient demographics and tumor characteristics were compared using the chi-square test. The HCC death rate was compared between groups using the Kaplan–Meier method. Risk factors for survival outcome were analyzed using multivariate Cox regression models. All statistical analyses were performed using the statistical software package SPSS for Windows, version 22 (IBM Corp, Armonk, NJ, USA). Statistical significance was set at two-sided *P* < 0.05.
